# Suicidal ideation, suicidal plans and suicide attempts in patients with chronic pain: a prospective qualitative research Study 1: 2011 – 2015 Study 2: 2015 - 2019

**DOI:** 10.1192/j.eurpsy.2024.364

**Published:** 2024-08-27

**Authors:** E. Z. Guertzenstein, M. J. Teixeira

**Affiliations:** ^1^ DIVISÃO DE NEUROCIRURGIA DO DEPARTAMENTO DE NEUROLOGIA DA FACULDADE DE MEDICINA DA UNIVERSIDADE DE SÃO PAULO; ^2^Departamento de Neurocirurgia, Divisão de Neurocirurgia da Faculdade de Medicina da Universidade de São Paulo, São Paulo, Brazil

## Abstract

**Introduction:**

The association among suicidal ideation, plans, attempts and pain has not attracted as much attention as the association between suicidal ideation and attempts and psychiatric disorder.

**Objectives:**

The aim of this prospective study was to establish if patients with chronic pain associated or not to psychiatric disorders with ideation and planning for a suicide attempt will aways end in a suicide act.

**Methods:**

The patients were initially examined through structured interview, scan-schedules for clinical assessment in neuropsychiatric – version 2.0 used only to diagnose. (HDRS – 17) - Hamilton Depression Rating Scale, 17 itens version, (HAMS) Hamilton Anxiety Rating Scale, (CGI) - Clinical Global Impression: (CGI – S) Severity of illness and (CGI – I) Clinical Global Improvemnt. Pain intensity through numerical rating scale. Those were repeated throughout the research.

Patients

Study 1 - 325 patients (244W, 81M aged 19 – 58) with chronic pain, suicidal ideation or plan associated or not to psychiatric disorders.

124 had chronic pain without psychiatric disorder. 54 suicidal ideation and 70 had suicidal plan.

201 had chronic pain associated with phychiatric disorders. 7 with opioid dependence after pain and suicidal plans. 4 with adjustment disorders before pain and suicidal ideation.

3 with somatoform disorder before pain and suicidal plan. 125 had chronic pain associated with: general anxiety disorder, mixed anxiety and depression, severe panic pain before or after pain with suicidal plans or ideation. 62 patients presented chronic pain associated with depressive disorder: recurrent severe depression without psychotic symptoms; moderate recurrent depression without psychotic symptoms. Before or after the occur of pain with suicidal ideation or suicidal plans.

Study 2 -132 patients remained in treatment. (79W and 53M aged 20 to 59)

54 had chronic pain without psychiatric disorder with plans or ideation suicidal. 78 Patients had chronic pain and a psychiatric disorder. 16 became dependent on opioids after pain and suicidal plans. 36 with chronic pain associated with anxiety disorder: general anxiety disorder before or after pain with suicidal plans. 26 had chronic pain associated with depressive disorder: recurrent severe depressive disorder with or without psychotic symptoms with suicidal ideation.

**Results:**

Study 1 - No patient attempted suicide. 54 patients with pain without psychiatric disorders considered suicidal thoughts absurd and intrusive.

Study 2 - No patient died. 51 attempted suicide

47 remained with suicidal ideation

33 did not have suicidal ideation or suicidal plans. They adapted their lives to chronic pain regardless of presenting a psychiatric illness.

**Conclusions:**

The authors concluded that a suicidal act is not always necessarily an expression of chronic pain associated/not with psychiatric disorder.

**Disclosure of Interest:**

None Declared

